# The Potential of β-Synuclein-Specific Regulatory T Cell Therapy as a Treatment for Progressive Multiple Sclerosis

**DOI:** 10.3390/ijms262311534

**Published:** 2025-11-28

**Authors:** Grace E. Osmond, Nevin A. John, Yi Tian Ting, Joshua D. Ooi

**Affiliations:** 1 Department of Medicine, School of Clinical Sciences, Monash University, Monash Medical Centre, Clayton, VIC 3168, Australia; 2 Department of Neurology, Monash Health, Clayton, VIC 3168, Australia

**Keywords:** autoimmunity, multiple sclerosis, translational immunology, regulatory T cells, Treg therapy, cell-based therapy, autoimmune disease, autoantigens, beta-synuclein

## Abstract

Disease progression in multiple sclerosis (MS) is now known to affect many patients, even those not diagnosed with progressive subtypes. Progressive and neurodegenerative aspects of MS are poorly treated by currently available therapies. Research on new therapeutic options is needed to improve health outcomes in people with MS. This review highlights the potential for treatment using an engineered T cell receptor–regulatory T cell (TCR-Treg) therapy targeting the presynaptic protein beta-synuclein. Tregs respond to self-antigens presented on human leukocyte antigen (HLA) class II with anti-inflammatory and pro-neural healing effects, but this response is impaired in MS patients. Since the HLA-DRB1*15:01 allele is known to contribute to MS pathogenesis, a TCR specific to a known antigen presented on DRB1*15:01 can be transduced into Tregs to direct them to activate within the inflamed brain tissue. Beta-synuclein is released from neurons at a high level after neural damage, may be presented on HLA, enables homing of specific T cells to the grey matter, and is immunogenic in progressive MS patients. This review presents beta-synuclein as a disease-relevant antigen to target for therapeutic development.

## 1. Multiple Sclerosis

Multiple sclerosis (MS) is a chronic neurological autoimmune disorder encompassing both inflammatory demyelination and neurodegeneration [1]. The MS Atlas estimates that 2.9 million individuals have been diagnosed with MS globally, and that this prevalence is increasing [2]. MS causes varied neurological symptoms depending on the affected part/s of the central nervous system (CNS), from optic neuritis resulting in visual changes and pain, to sensory, motor and autonomic symptoms resulting from damage to the spinal cord [3]. Cognitive impairment, mood disorders (notably depression), sleep disorders and fatigue may also occur. These symptoms can be debilitating and reduce an individual’s ability to work and live independently [4], and as such, living with MS reduces quality of life [5]. MS diagnosis is also associated with a 7 to 10-year reduction in life expectancy [6,7].

Multiple sclerosis is a heterogeneous disease with several diagnostic subcategories. Around 85% of MS patients are first diagnosed with relapsing–remitting MS (RRMS), defined by multiple distinct relapses with clinical symptoms and lesions confirmed by magnetic resonance imaging (MRI) [3]. The remaining 15% of patients are first diagnosed with primary progressive MS (PPMS). Adding to the proportion of patients who are diagnosed with progressive MS, many RRMS patients will transition to secondary progressive MS (SPMS) over time. Specific figures vary, with one study finding 18.1% of RRMS patients transition to SPMS [8]. Another study used objective classifiers to reassess patients with clinically assigned RRMS or SPMS. After re-classification, the proportion of patients with SPMS tended to increase to 15.1–58%, varying with the classifier used [9]. Natural history studies in untreated cohorts found the vast majority of RRMS patients would transition to SPMS within 20 years from diagnosis, but median time to progression to SPMS has been substantially increased by treatment with disease-modifying therapies (DMTs), thereby reducing the proportion of patients that enter SPMS [10,11]. Recent research has proposed that progression independent of relapse activity (PIRA) occurs in patients across all subtypes and may be the main contributor to disease worsening even in RRMS [12]. As opposed to relapse-associated disease worsening, which is treated well by current therapies, PIRA lacks targeted treatment [13]. Diagnosing and treating progression is emerging as a key priority.

## 2. Predispositions and Aetiology of MS

Genetic and environmental factors impact MS predisposition, with one estimate finding genes account for 22.4% of susceptibility [14]. A recent genome-wide association study of MS patients found 233 gene variants associated with MS, with 32 of these located within the human leukocyte antigen (HLA) locus [15]. HLA genotype, and specifically presence of the HLA-DRB1:15*01 (DR15) allele, correlates with many autoimmune diseases including MS, Goodpasture’s syndrome [16], ulcerative colitis [17], and systemic lupus erythematosus [18]. Carriage of the DR15 allele is the single most strongly associated genetic risk factor to development of MS, with an odds ratio of 3.17 in the Caucasian population [19]. Accordingly, the prevalence of DR15 in MS patient populations is higher than in healthy controls, with several studies reporting that over 50% of cases carry this allele [20], a substantial increase from the estimated 14.51% of the European-American population [21]. In non-European populations, other HLA-DRB1 alleles including 04:05 and 15:03 may be more strongly associated with MS than 15:01 [20,22].

It is theorised that the association of HLA-DR15 with MS is primarily due to its role in antigen presentation to T cells and this allele’s unusual propensity to present self-peptides [23]. This mechanism is also supported by the distinct peptide repertoire of HLA-DR15 [24]. However, recognition of the HLA molecule itself may also contribute. The helices of the HLA peptide-binding groove can make up the majority of the T cell receptor (TCR) contact surface on HLA-peptide [25] and HLA surface features are known to contribute to autoreactivity in other diseases [26]. Self-peptides derived from digestion of HLA-DR15 molecules also form part of the immunopeptidome due to normal immune surveillance and cross-presentation, which is known to contribute to multiple sclerosis [27,28]. The relative contribution of each of these aspects to DR15′s disease association is not yet clear.

Other immune abnormalities contribute to the development of autoimmunity seen in MS. Non-HLA genetic associations include those involved in cytokine signalling such as the IL-2 receptor, vitamin D metabolism, immune cell differentiation and activation, and expression or splicing of other predisposing genes [29,30]. Expression of MS susceptibility-associated genes is enriched in both adaptive and innate immune cell types, including T and B cells, dendritic cells, and microglia, as well as in thymic tissue [15]. Specifically, epigenetic studies have identified B cells and microglia as important in disease development [31]. While immune-associated genes appear to control predisposition to MS, it has been proposed that variants in regulation of genes expressed in oligodendrocytes and neurons more strongly impact the rate of disease progression [32].

Epstein–Barr virus (EBV) is the most strongly associated environmental risk factor, with risk of developing MS increasing by 32-fold in individuals seropositive for antibody against EBV nuclear antigens [33]. The causal link has not been fully confirmed due to incomplete understanding of possible mechanisms, but a molecular mimicry mechanism based on recognition of EBV antigens presented on DR15 and development of a cross-reactive response to myelin and other neural antigens has been proposed to contribute, alongside changes to B cells due to EBV infection [34,35]. Several other environmental and lifestyle factors impact the likelihood of MS developing and the subsequent disease course, including geographic location, vitamin D level, obesity during childhood, and smoking status [1,36].

## 3. Immune and Degenerative Pathological Mechanisms

The pathology of MS is defined by an autoimmune response to myelin and other CNS antigens resulting in high levels of immune cell infiltration, plaque-like areas of demyelination and damage to neurons (often surrounding a blood vessel), and diffuse damage to both grey and white matter including the optic nerve and spinal cord [3]. RRMS tends to show greater white matter pathology and is typified by defined lesions, while progressive MS (PMS) tends to show greater damage to the cortical and deep grey matter, diffuse neurodegeneration predominates with rare or no defined white matter lesions, and meningeal inflammation is often present [37]. The blood–brain barrier, while considered to be abnormally permeable in RRMS allowing the migration of reactive lymphocytes to the CNS, is less so in PMS, reflecting compartmentalised inflammation [38].

The underlying processes of MS can be described as a continuum where many patients display both neurodegeneration and active inflammatory responses [37]. Debate is ongoing about which is the primary disease initiator [13] but it is clear that both contribute to pathology and symptoms and likely form a cycle in which neurons are damaged, antigens are released, and immune reactivity to those antigens leads to further neural damage [1,39]. Non-inflammatory neural damage in PMS is contributed to by numerous processes including excitotoxicity, oxidative injury, mitochondrial failure, iron accumulation, and axonal degeneration [37,40]. However, the efficacy of B cell depletion with ocrelizumab in PPMS indicates inflammatory autoimmune processes still occur in progressive subtypes [41].

Local glial cell subsets also contribute to pathogenesis. Dysregulated expression of many genes in glial cells occurs under inflammatory conditions in MS, which may reflect cell stress and/or pathological involvement [42]. PIRA may partly be caused by impaired or absent oligodendrocytes failing to remyelinate neurons, meaning that neural conduction remains impaired within inactive plaque areas [43]. Activated macrophages/microglia also contribute to chronically active lesions, by impairing healing and in some cases forming slowly expanding lesions [37]. As antigen-presenting cells, microglia and astrocytes also contribute to activation of adaptive immune cells, especially CD4+ T cells [39].

While CD8+ T cells contribute to cell death, dysregulated CD4+ T cells, notably Th1 and Th17, also strongly influence neuroinflammation in MS [44], secreting inflammatory cytokines and participating in tertiary lymphoid follicles within the submeningeal space alongside B cells [38]. The strong association with HLA type and consistent findings of expanded self-reactive T cell subsets make clear that recognition of antigens via T cell receptor (TCR) is key for MS pathogenesis [45].

Consistent failure to identify ubiquitous antigens among MS patients supports the understanding of MS as a heterogeneous disease [46,47,48]. Significant variance in T cell target antigens exists between patients and multiple antigens are immunogenic within each patient [48]. Oligoclonal IgG bands are present in the cerebrospinal fluid (CSF) of the majority of MS patients as a result of B cells producing antibodies against cellular or intracellular antigens [49] with one study identifying 166 such autoantigens targeted by at least 10% of clinically isolated syndrome patients [47].

Finally, ectopic lymphoid follicles were found in the meninges of 40% of SPMS cases in one study and were associated with earlier death and more aggressive progression [38]. Meningeal inflammation may be diffuse or organised into follicles and is often more intense deep within sulci where it co-locates with stronger cortical damage [39].

## 4. Grey Matter Damage and Its Importance in Pathology

Grey matter damage and loss is known to be a significant driver of disease. It is correlated with several key symptoms and is more prominent in PMS than RRMS [50]. White and grey matter damage appear to be linked in extent but not necessarily causatively related, and less strongly correlated in PMS than RRMS [39]. Damage to grey matter occurs as a result of numerous independent but cooperative processes, both immune and non-immune, as discussed above. Cortical lesions are primarily mediated by microglia rather than lymphocytes, but are often associated with meningeal inflammation, which may be via direct inflammatory damage or microglial activation due to secreted factors [38]. Cortical damage can also be a downstream effect of inflammatory white matter lesions via axonal degeneration [39].

Cortical and deep grey matter lesions and volume loss have a stronger correlation with disease progression, worse cognitive function, and epileptic seizures than white matter lesions [51,52,53]. Similarly, total grey matter volume loss appears to be predictive of development of MS from clinically isolated syndrome, and progression to SPMS from RRMS [54]. Submeningeal or cortical lesions are more difficult to detect using MRI techniques than classical white matter lesions, so their contribution to disease is likely underestimated [37,54]. The partial independence of grey and white matter damage, and grey matter’s disproportionately large effect on disease progression in PMS patients, suggest targeting grey matter may be a viable therapeutic strategy.

## 5. Current Disease-Modifying Therapies

Progression eventually affects most individuals diagnosed with MS. Despite this, few therapies are available and the poor understanding of pathological mechanisms specific to PMS and lack of sensitive biomarkers hampers development of new therapies [55]. Treatment of MS may take the form of symptomatic treatment, or of treatment with disease-modifying therapies (DMTs) which aim to resolve underlying pathology. RRMS patients have access to a range of approved medium- or high-efficacy DMTs which are primarily immunomodulatory or immunosuppressive [3]. These DMTs have generally failed to show efficacy in reducing disease progression in PMS [56,57], suggesting the pathological mechanisms are significantly different.

Active SPMS (with relapses alongside continuous disease progression) can be treated with sphingosine-1-phosphate (S1P) receptor modulators [58]. S1P has roles in processes including immune cell migration and survival/activation of neurons, oligodendrocytes, and microglia, and the antagonism of its receptor reduces circulating lymphocyte counts and contributes to neuroprotection [59]. Also available are interferons, immunosuppressive cytokines that impair T and B cell proliferation and function and are more effective in patients with active SPMS than non-active [60,61]. Some guidelines allow use of immunosuppressive DMTs such as ocrelizumab, cladribine and mitoxantrone for patients with active SPMS [60].

PPMS has only one approved DMT, ocrelizumab, an anti-CD20 antibody which depletes pre-B, mature and memory B cells [62,63]. The ORATORIO trial, a Phase 3 randomised controlled trial in PPMS patients found ocrelizumab modestly reduced disability progression, brain lesion size and rate of total brain atrophy [63]. Further analysis of this trial along with an expanded patient cohort suggests that the efficacy did not differ between active and non-active PMS patients [64] which was supported by a later observational study [65]. It is still unclear how ocrelizumab addresses progressive pathology. Its mechanism of nonspecific immunosuppression also slightly increases infection and cancer risk [63].

From a mechanistic perspective, impairment of immune cell migration to the brain does not address pathology in progressive MS due to compartmentalised inflammation, and immunomodulatory treatments fail to address non-inflammatory processes of neurodegeneration resulting in poor treatment of patients with non-active progressive disease [66]. Patients with non-active progressive disease are less likely to be receiving treatment and see less benefit from most available therapies [60,67]. Taken together, there is a relative paucity of treatments addressing disease progression in MS representing an area of great unmet need [57].

## 6. Clinical Trials and Proposed Therapies for Progressive MS

Significant research is being undertaken to develop therapies for progressive MS (Table 1). Susin-Calle et al. identify three key themes in recent phase 2 trials of modulating the immune response, supporting neuroprotection and promoting remyelination [68]. Several therapies aimed to induce tolerance or reduce inflammation: ATX-MS-1467, a tolerogenic myelin peptide vaccine, and foralumab, an antibody targeting CD3 with the aim of suppressing inflammatory T cells and inducing tolerogenic regulatory T cells (Tregs). Inflammatory cells are targeted by limiting T and B cell expansion (vidofludimus calcium) or inhibiting their function via receptor antagonism (frexalimab, which has moved to phase 3 trials in NCT06141486, and obexelimab), or direct killing of inflammatory cells via chimeric antigen receptor (CAR)-T cells (KYV-101).

Modification of existing anti-inflammatory therapies is also a major theme in current trials. Cladribine, a DNA synthesis inhibitor that impairs immune cell replication and is currently used in RRMS, was trialled in advanced PMS and has now moved to phase 3. One trial attempted to apply current treatments for RRMS, rituximab and glatiramer acetate, to SPMS (NCT03315923) with mixed results [76]. Aspects of ocrelizumab treatment and response in PMS patients are examined in phase 3 trials which have recently published some results [69,70,71].

Several current trials are assessing inhibitors of Bruton’s tyrosine kinase (BTKi), described in more detail by Naydovich et al. [74]. Notably, the HERCULES trial (NCT04411641) has recently published results indicating efficacy in non-active SPMS, with reduced disease progression compared to placebo [75]. BTKi drugs aim to reduce B cell development and macrophage/microglial activation, targeting B cell-mediated inflammation and slowly expanding lesions (likely explaining its efficacy in PMS patients), and protective effects on myelin have also been proposed but lack data [86]. BTKi drugs also show good CNS penetrance, enabling them to address compartmentalised inflammation [87]. Masitinib, another tyrosine kinase inhibitor that alters innate cell function and may have neuroprotective effects [88], is in a phase 3 trial for PMS (NCT05441488) which aims to use more comprehensive outcome measures to validate success in previous phase 3 trials in non-relapsing PMS.

Neuroprotective agents in recent phase 2 trials include ibudilast, which appeared to reduce brain atrophy and slowly expanding lesions in NCT01982942, but no phase 3 trial is scheduled, and lipoic acid which is proposed to both protect against tissue damage and reduce inflammation. Remyelinating agents include several drugs to protect oligodendrocyte survival or induce oligodendrocyte maturation (SAR443820, clemastine, and metformin), and bazedoxifene, an oestrogen receptor modulator to induce myelin repair [68].

Proposed therapies with the specific aim of addressing neurodegeneration reflect the diverse mechanisms at work in PMS, extending from the focus on immune suppression to include immune-independent aspects of disease such as cellular metabolism changes, glial cell function, coagulation factors, blood–brain barrier function, and induction of immune tolerance [89]. In addition, many investigative therapies attempt to modify processes of neurodegeneration and neural healing, such as oxidative damage, necroptosis, remyelination, extracellular matrix repair and others that underlie disease progression outside of inflammatory relapses [90,91].

Endpoints used to assess treatment efficacy in clinical trials on PMS populations assess a variety of typical pathological features including disability progression (measured using functional assessment scales and practical tests), measurements via MRI of slowly expanding lesions and atrophy, serum neurofilament light chain (a marker of neural death) and glial fibrillary acidic protein (a marker of astrocyte activity, used to assess neurodegeneration), inflammatory cell counts, and microglial activation (measured using positron emission tomography) [68]. However, due to the lack of confirmed biomarkers, functional assessment using the Expanded Disability Status Scale remains the most commonly used endpoint, as it is the most robust standardised measurement of a patient’s clinical disease available for the PMS setting.

Cell therapy interventions for PMS in current/upcoming clinical trials (Table 2) focus on stem cell therapies where one small phase 1 study showed promising results [92]. T cell therapies are also currently being investigated, including cytotoxic T cell-based therapies to deplete B cells, and one engineered T cell receptor–regulatory T cell (TCR-Treg) therapy targeting an undisclosed myelin-associated antigen, ABA-101 [93]. While all these approaches are immunomodulatory, with the anti-B cell therapies being directly immunosuppressive and hematopoietic stem cell transplant after immune cell ablation aiming to reconstitute the immune system, mesenchymal stem cell therapies may be both anti-inflammatory and neuroprotective [92]. Being Treg-based, ABA-101 also promotes both immune tolerance and tissue healing.

## 7. Regulatory T Cells’ Anti-Inflammatory and Neuroprotective Functions

**Figure 1:**
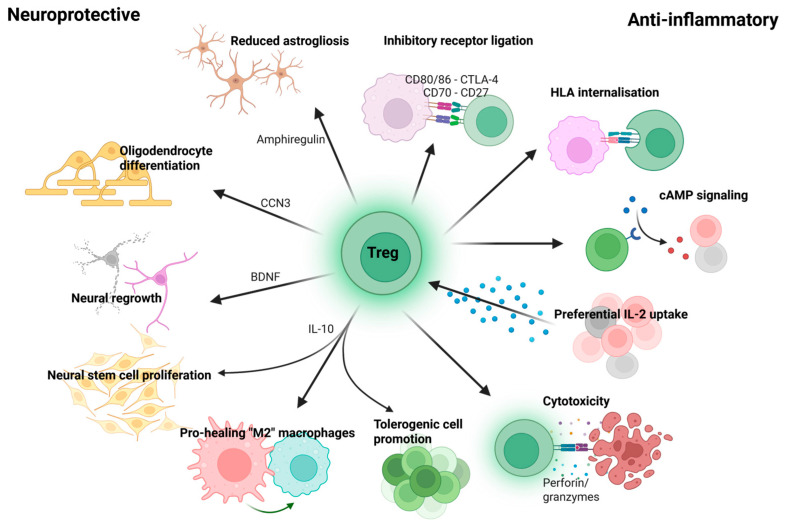
Anti-inflammatory and neuroprotective effects of Tregs that may be beneficial in treating MS [98]. Tregs’ neuroprotective effects are mediated by secretion of amphiregulin which protects against astrogliosis, CCN3 which promotes oligodendrocyte differentiation and thus supports remyelination, and BDNF which promotes neural growth. Regarding anti-inflammatory effects, inhibitory receptor ligation reduces activation and costimulatory ability of APCs. Similarly, antigen-dependent HLA internalisation from APCs reduces antigen presentation to inflammatory cells. Preferential IL-2 uptake and cAMP signalling result in death of inflammatory cells, while Tregs can also directly lyse inflammatory cells with perforin and granzymes. Secretion of IL-10 contributes to many benefits including promoting neural stem cell proliferation, contributing to a pro-healing macrophage phenotype, and proliferation of tolerogenic cells including Tregs.

Regulatory T cells (Tregs) are an anti-inflammatory CD4+ T cell subset, contributing to immune homeostasis by preventing excessive response to self or foreign antigens. The thymically generated Treg TCR repertoire is predominantly self-antigen specific as a result of exposure to self-antigens during their development. Tregs can also be generated in response to innocuous food, commensal microbe, or environmental antigens given an appropriate TCR exists within the population. This receptor enables Tregs to migrate to specific tissues in which these antigens are present, where they suppress inflammatory effector cells through numerous contact-dependent and non-contact-dependent mechanisms (Figure 1) [96,97].

Contact-dependent mechanisms depend on receptor-ligand binding to suppress inflammatory cells. Inhibitory ligands are expressed on the surface of Tregs, which induce suppression of antigen-presenting cells (APCs) on contact. One such ligand, CTLA-4, induces quiescence in T cells and is associated with contraction after an infection, as well as triggering internalisation of costimulatory molecules on antigen-presenting dendritic cells (DCs) [99,100]. On contact between Treg-expressed CTLA-4 and DC-expressed CD80/CD86, as well as Treg-expressed CD27 and DC-expressed CD70, the DC’s receptors are internalised, limiting its ability to activate naïve inflammatory T cells [101,102]. Tregs can also bind to and internalise HLA on APCs’ surface via TCR-dependent recognition of the HLA-peptide complex, limiting the activation of inflammatory T cells recognising that same peptide [96,103]. When applied to a TCR-Treg therapy, these mechanisms may allow suppression of beta-synuclein (βsyn)-dependent inflammatory T cell responses indirectly, by suppressing local antigen-presenting cells. The non-antigen-dependent effects may also contribute to generalised suppression of inflammatory T cell responses not targeted at βsyn.

Non-contact-dependent mechanisms utilise secretion and uptake of soluble mediators and contribute to maintaining an immunosuppressive microenvironment and broader homeostatic effects. Tregs contribute to suppression of effector T cells by uptake of IL-2 via the high-affinity receptor CD25, thereby reducing availability of a necessary activatory and survival signal for CD8+ T cells, NK cells and type 2 ILCs [96]. They also induce suppressive c-AMP signalling via ectoenzymes which produce adenosine; and direct transfer through gap junctions [104]. Tregs can also directly kill inflammatory effector cells (including B cells) via perforin/granzyme mediated cytotoxicity [105,106]. All of these effects, in a therapeutic context, may limit local pathological T cell and innate immune cell activity. Finally, Tregs promote development of further tolerogenic cell populations in the periphery, such as tolerogenic DCs and regulatory B cells by secreting IL-10, IL-35, and TGF-β, among other mechanisms [107]. This IL-10 release also acts in an autocrine manner resulting in a feedback loop of expanding Treg populations [108,109]. Stimulation of downstream tolerogenic proliferation supports expansion and maintenance of the immunosuppressive effect initially induced by TCR–Treg infusion and localisation. These diverse suppression mechanisms mean Tregs can effectively address both cellular and humoral inflammatory pathology, making them suitable to address the complex immunopathology of MS.

Tregs are also known to be neuroprotective (Figure 1). Activated Tregs migrate to the inflamed brain through chemokine gradient migration using the receptors CCR6, CCR8 and LFA-1, but require TCR recognition of self-antigens to activate [110,111,112,113,114]. Damage to the blood–brain barrier is known to increase ease of cell migration into the brain, but Tregs are also capable of activation and proliferation once inside the brain, demonstrated by high prevalence of a small number of TCR sequences in CNS Tregs responding to a monophasic experimental autoimmune encephalomyelitis (EAE) attack [115]. Activated thymic-derived Tregs support tissue remodelling after acute spinal cord injury by resolving inflammation mediated by adaptive immune cells, preventing excessive inflammation and damage to myelin and mediating a return to homeostasis [116].

Importantly, Tregs can directly contribute to neural healing independent of their function in regulating inflammatory lymphocytes [56]. Tregs have been found to promote oligodendrocyte differentiation and remyelination by expression of CCN3 [117], polarise local macrophages/microglia toward an anti-inflammatory pro-healing phenotype by releasing IL-10 [118], and secrete amphiregulin to limit astrogliosis (pathological overactivation of astrocytes [119]) and thereby promote neural survival and neurological function in the chronic phase of injury in stroke models [110]. A TCR-Treg therapy thus has the potential to directly support remyelination and act against pathological microglia and astrocyte activity seen in MS. Tregs also contribute directly to neural growth and healing by releasing BDNF, a neural growth factor [120], and stimulating neural stem cell proliferation via IL-10 [121]. Both of these factors may aid in neural regrowth in atrophied areas and inactive lesions within the MS brain, although significant further research is required to transition between in vitro effects of secreted factors and in vivo neural tissue healing activity. The neuroprotective functions of Tregs are still not fully understood but research so far supports their benefits in maintaining health and in the treatment of neuroinflammatory diseases [122].

## 8. Treg Dysfunction in MS

Autoimmune diseases can result from breaches in tolerance due to loss of suppression of autoreactive cells by Tregs, which may be due to reduced number or function [123]. This Treg dysfunctionality has been noted as a driver of MS pathogenesis [124]. The relevance of Treg number to MS pathogenesis is debated, with a meta-analysis finding no statistically significant change in overall Treg abundance among eight studies [125] but one later study finding peripheral Treg level to be reduced in MS patients compared to control, and further reduced in relapses than remission within the same patient [126]. In contrast, another study found the number of activated Tregs in MS to be higher than in healthy patients, with levels increasing with time since diagnosis and with time since last relapse, but with impaired function [127].

Functional changes found in MS patients’ Tregs include low CCR6 expression reducing their ability to migrate to the brain [127], reduced IL-10 production impairing their suppressive capacity [128], and increased expression of the apoptosis-inducing receptor Fas [129,130]. Tregs in MS also display an unusually inflammatory phenotype including expression of interferon-gamma, along with signalling abnormalities [131]. In addition, the balance of Th17/Treg subsets is impaired in many autoimmune diseases, including MS [132]. Effector T cells also display resistance to Treg suppression, likely mediated by IL-6 [133], which is addressed by some DMTs [134,135]. However, animal data indicates Tregs retain some protective capacity, as abrogating their function worsens disease [136]. Lower Treg number and function in autoimmune diseases can be rescued by ex vivo expansion [126], making autologous expanded Tregs a viable therapy for these patients [137,138].

## 9. Therapeutic Use of Tregs

Restoration of Treg number or function can redress imbalances in immune homeostasis. Eggenhuizen et al. outline several approaches to this goal [139]; polyclonal Treg development may be stimulated by chemical supplementation of cytokines, or exposure to harmless antigens via microbiome therapy. More targeted therapies to induce an antigen-specific Treg response include ‘tolerogenic vaccination’ with a peptide or protein antigen or tolerogenic DC therapy.

Polyclonal or antigen-specific Treg populations can also be directly manipulated. Tregs can be induced from peripheral CD4+ T cells [140] or a patient’s preexisting Tregs can be expanded and stimulated, with or without transduction of a chimeric antigen receptor (CAR) or recombinant TCR to confer antigen specificity [141]. Antigen specificity is beneficial for therapeutic Tregs, as it localises and prolongs the anti-inflammatory response, reduces potential off-target effects, and appears to reduce the dosage required for effective immunosuppression [56,139]. In contrast to TCR-T cell therapies, which use the inherent ability of TCRs to recognise HLA presenting an antigen, CAR-T cells use a variety of other approaches that are generally not HLA-restricted, notably binding domains derived from antibodies [142]. While CARs are capable of recognising antigens in native form, an antigen-specific TCR would be most appropriate to address HLA-DR15-dependent antigen responses in MS.

Regarding the choice of target antigen, Tregs’ capacity to induce bystander suppression means that an inflammatory response can be suppressed by Treg targeting of an antigen that is not disease-relevant but is present at a high concentration in the inflamed area. This approach has found some success both experimentally (targeting EAE triggered by myelin oligodendrocyte glycoprotein or proteolipid protein with myelin basic protein (MBP)-specific Tregs [143,144]) and in clinical trials (targeting Crohn’s disease with polyclonal ovalbumin-specific Tregs, in conjunction with dietary supplementation of ovalbumin to enable Tregs to activate at the inflamed gut membrane [145]). These results indicate that localising and activating the Tregs may be sufficient for therapeutic efficacy, regardless of the strength of a patient’s inflammatory response to that antigen and including antigens that are not immunogenic but presented by APCs as part of normal immune surveillance. In the MS context, this indicates a grey matter antigen can be targeted to address grey matter pathology. This also improves generalisability of the proposed therapy, as although MS is known to have multiple antigenic targets, varying within and between patients, such a therapy could be applied to any patient with the recognisable HLA.

Tregs have been proposed as a therapy for various autoimmune and neurodegenerative disorders [146]. In Alzheimer’s disease, proposed benefits include reducing neuroinflammation, modulating microglial activation, reducing astrogliosis and reducing protein accumulation typical to Alzheimer’s disease [147]. Similarly, Treg therapies have been proposed for MS [56,148,149], incorporating the known immunomodulatory and neurological benefits of Treg therapies with targeting well-known antigens relevant to MS pathogenesis such as MBP [56]. Some existing treatments for MS affect Tregs, and proposals to alter the function or increase proliferation of Treg cells via specific cytokine, chemokine and small molecule drugs have been successful in the EAE model [130]. Likewise, autologous hematopoietic stem cell transplantation, a therapy being trialled for MS which aims to ablate and reconstitute the immune system to return to a less inflammatory state, also results in improved Treg number and function, which may contribute to its efficacy [150].

One small phase I trial of polyclonal Tregs in RRMS patients indicated they may prevent relapse and disability progression, but efficacy was dependent on the route of administration, with intrathecal administration being far more effective than intravenous [151]. The authors suggest this shows that MS is more complex than a simple systemic lack of Tregs and reflects benefits of localised immunosuppression [151]. Antigen-specific Tregs’ migration and local activation capabilities may confer the benefits seen in intrathecal administration while allowing use of less invasive intravenous administration. The current TCR-Treg trial discussed earlier, ABA-101, uses an engineered TCR recognising a myelin antigen and in vivo model data indicates successful tissue localisation and anti-inflammatory effect [93]. A study into the persistence of autologous expanded Tregs in type 1 diabetes found that up to 25% of the administered Tregs persist for over a year post administration, indicating that a Treg therapy may also provide enduring relief in MS [138].

Treg therapies for MS are cutting edge and some limitations and risks must be considered. These include instability of the Treg phenotype under inflammatory conditions, lack of information on the most effective dose and route of administration, confirmation that infused Tregs can migrate to the brain [124], poor knowledge of markers to identify and isolate the most effective and stable subsets, risks associated with genetic modification and long-term persistence of genetically modified cells, potential excessive or off-target immunosuppression, resistance of effector T cells to Treg suppression [152], cost and difficulty of production, and incomplete understanding of the best disease-relevant antigens to target with a TCR-Treg [56,148]. Methodological and safety concerns have been substantially addressed by previously successful Treg therapy trials [16,141,146,153,154,155,156,157]. Some concerns are unique to the proposed therapy, such as ensuring effective migration past the blood–brain barrier and ensuring effective immunosuppression despite the known resistance of inflammatory effector cells and compartmentalised inflammation. To address these issues, further studies are needed before such a therapy can reach clinical use. However, the potential benefits of a multivalent therapy addressing both inflammation and neurodegeneration in the undertreated PMS population support further development in this area. Here we propose beta-synuclein as a candidate target antigen.

## 10. Beta-Synuclein

βsyn is a small protein expressed within the cytosol of neurons in the CNS [158] and retinal cells [159]. βsyn is present in higher concentrations in grey matter than white, as it localises to the presynaptic terminals [160] where it may act as a chaperone for synaptic vesicles [161,162]. It is part of the synuclein family, along with the better-known alpha-synuclein, which is involved in a range of diseases known as synucleinopathies, including Parkinson’s disease and dementia with Lewy bodies [163]. Βsyn has been suggested to act neuroprotectively against alpha-synuclein pathology, inhibiting its binding to synaptic vesicle membranes, and inhibiting aggregation of alpha-synuclein in Parkinson’s disease [164,165]. In recent years research has expanded on βsyn’s role outside its relationship with alpha-synuclein, implicating it in synaptic function, dopamine uptake, apoptotic regulation and autophagy [166,167].

βsyn is indicated to have a role in cognition by several observations. Certain mutations may predispose one to developing Lewy body dementia and, in animal models, correlate more strongly with memory than motor impairment [168]. This may indicate a role for βsyn in maintaining synaptic function. Within the CNS, βsyn was found in aggregates in brain tissue isolated from individuals with Parkinson’s disease and Lewy body dementia [169] as well as in axonal spheroids in an inherited neurodegenerative disorder [170]. It is unclear whether these diseases and their associated cognitive deficits are a direct result of changes in βsyn level, location or function, as a result of changed ratio or interactions between βsyn and alpha-synuclein, or whether βsyn release or aggregation is a downstream effect [161]. No evidence exists for an active causative role of βsyn in MS.

## 11. Immune Presentation, Recognition and Response to βsyn

The initiation of an inflammatory adaptive immune response is dictated by antigen availability, presentation to inflammatory cells and capacity of those immune cells to recognise antigen in part or in whole. While βsyn is a cytosolic protein, numerous aspects contribute to increasing its availability to APCs. Release of βsyn into the extracellular environment occurs even under healthy conditions due to the dynamic membrane environment at the synapse and has been proposed to cause autoimmunity [171]. βsyn is released at higher levels under conditions of synaptic damage and diffuses from brain tissue into CSF and blood, which has led to the proposal to use βsyn as a biomarker for various chronic neurodegenerative diseases or conditions of brain damage, including prion diseases [172,173,174].

In an acute context, high blood βsyn has also been shown to predict poor outcomes from traumatic brain injury and stroke [175,176]. Plasma βsyn peaks early after traumatic brain injury and declines over the following 10 days after hospital admission, suggesting it is released immediately on synaptic damage and release then slows because the synaptic degeneration is not continuous [176]. In contrast, in the MS context where the brain undergoes repeated and chronic inflammatory damage, this finding implies βsyn release may be continuous, promoting development of an inflammatory response.

Research on synaptic protein levels in brain tissue and CSF in neuroinflammatory diseases is inconclusive. Short term increased levels are thought to result from synapse damage, but long-term decreased levels occur as a result of widespread synapse loss that is associated with cognitive deficits, and of uptake by immune cells [177,178]. Barba et al. investigated levels of synaptic proteins in MS, including βsyn, and determined CSF βsyn levels were decreased in MS patients in comparison to other neurological diseases, and that decreasing βsyn levels were associated with increasing cognitive impairment and lower brain/thalamus volume; the explanation for this is still unknown [178]. Expression levels may also change in neural diseases [167]. However, due to the countervailing processes that may impact synaptic protein release and uptake, the total concentration of βsyn in CSF may be disconnected from its availability to APCs within the brain and whether an immune response can occur.

Exploring sequence homology and binding affinity to HLA between peptides associated with MS can indicate whether their presentation may occur in the pathogenesis of MS. Sequence-similar peptides derived from Epstein–Barr nuclear antigen 1 and βsyn bound with similar affinity to a known immunogenic MBP peptide into HLA DR2b (DRB1*15:01, DRA1*01:01) [179]. This indicates that βsyn can be presented by APCs in MS patients with the DR15 allele, and provides more evidence for the molecular mimicry hypothesis whereby, during EBV infection, cross-reactive TCRs are generated to its nuclear antigens that also respond to βsyn, prolonging inflammation and resulting in the development of MS. However, this hypothetical pathway to βsyn reactivity has not yet been confirmed by structure or patient data.

Βsyn is encephalitogenic in rats, where immunisation with βsyn induced EAE and caused epitope spreading resulting in reactivity to myelin antigens [180]. This study also found that βsyn-specific T cells were sufficient to transfer passive EAE, while sera (containing βsyn-specific antibodies) were not, reflecting the importance of the HLA-TCR axis in disease. A later study found that microglia from the rat grey matter elicited a stronger response in T cells reactive to βsyn than microglia from the whole brain, potentially because they present more βsyn [181]. Polyclonal βsyn-specific T cells also localised to the grey matter, suggesting that Tregs with this specificity show promise to address grey matter pathology.

To determine whether reactivity to βsyn forms part of MS pathogenesis, Lodygin et al. also explored the immunogenicity of βsyn in MS patient blood, finding inflammatory T cells reactive to βsyn significantly increased in SPMS and PPMS patients when compared to both healthy controls and patients with RRMS [181]. These results provide the basis for development of an antigen-specific Treg therapy targeting βsyn for PMS.

## 12. Conclusions

MS is a complex disease comprising both neuroinflammation and neurodegeneration. The persisting lack of effective therapies to address progression in MS, despite progression occurring in many MS patients across subtypes and the overall increasing prevalence of MS worldwide, is an issue requiring urgent research. Progression currently represents a permanent loss of ability for these patients with little to no capacity to regain function. Treg therapies have potential to address both inflammatory and neurodegenerative aspects of MS, to slow or stop disability progression, and contribute to neural healing and corresponding improvements in functional capacity. A DR15-βsyn specific Treg therapy may address the grey matter pathology that is associated with progression and cognitive function impairment. Beyond the direct application of this research to produce a therapy, investigating the efficacy of βsyn-targeted suppression would expand the understanding of MS pathogenesis and DR15-dependent autoimmunity.

## Figures and Tables

**Table 1:** Current and recent phase 2 and 3 clinical trials for PMS, excluding cell therapies.

Drug Class	Drug Name/Subclass	Clinical Trial ID	Phase	Study Completion Year	Publication References
Immunosuppressant monoclonal antibody	Ocrelizumab	NCT03606460	3	2019 *	[69]
		NCT02688985	3	2023 *	[70]
		NCT03691077	3	2024 ^?^	
		NCT05974839	3	2024 *	
		NCT05232825	3	2025 *	[71]
		NCT03523858	3	2026	
		NCT04548999	3	2027	
		NCT04035005	3	2028	
	Ocrelizumab and rituximab	NCT04688788	3	2029	
	Masitinib	NCT05441488	3	2028	[72]
	Frexalimab	NCT04879628	2	2027	[73]
		NCT06141486	3	2028	
	Obexelimab	NCT06564311	2	2026	
	Foralumab	NCT06292923	2	2025	
Tolerogenic peptide vaccine	ATX-MS-1467	NCT01973491	2	2016 *	
Bruton’s tyrosine kinase inhibitor [74]	Tolebrutinib	NCT04742400	2	2025	
		NCT04411641	3	2024 *	[75]
		NCT04458051	3	2025	
		NCT06372145	3	2029	
	Fenebrutinib	NCT04544449	3	2027	
DNA synthesis inhibitor	Cladribine	NCT04695080	2/3	2027	
		NCT05961644	3	2027	
	Vidofludimus calcium	NCT05054140	2	2025	
Multiple	Rituximab and glatiramer acetate	NCT03315923	2/3	2019 *	[76]
Neuroprotective	Ibudilast	NCT01982942	2	2017 *	[77,78,79,80]
	Lipoic acid	NCT03161028	2	2024 *	[81]
	Intranasal insulin	NCT02988401	1/2	2021 *	[82]
	Simvastatin	NCT03896217	2	2023 *	
		NCT03387670	3	2024 *	[83]
	SAR443820	NCT05630547	2	2024 ^t^	[84]
	Metformin and clemastine	NCT05131828	2	2025	
	Bazedoxifene	NCT04002934	2	2025 *	[85]

* completed, ^t^ terminated, ^?^ unknown status.

**Table 2:** Clinical trials involving cell therapies for progressive MS.

Drug Class	Drug Name/Subclass	Clinical Trial ID	Phase	Study Completion Year	Publication References
Stem cell	Autologous hematopoietic stem cells	NCT06900192	1	2029	
		NCT04047628	3	2029	
	Mesenchymal stem cells	NCT06360861	1	2024 *	[92]
		NCT06592703	1	2029	
	Intrathecal amniotic fluid stem cells	NCT06841068	1/2	2026	
	Autologous stromal cells	NCT06961383	2	2028	
Anti-CD19 chimeric antigen receptor (CAR)-T cell [94]	KYV-101	NCT06451159	1	2027	
		NCT06138132	1	2027	
		NCT06384976	2	2029	
	YTB323	NCT06675864	1/2	2030	
	CC-97540	NCT06220201	1	2027	
Anti-BCMA CAR-T cell [94]	CT103A	NCT04561557	1	2027	[95]
Regulatory T cell	ABA-101	NCT06566261	1	2027	[93]

* completed.

## Data Availability

No new data were created or analyzed in this study. Data sharing is not applicable to this article.

## Abbreviations

The following abbreviations are used in this manuscript:

MS	Multiple sclerosis
MRI	Magnetic resonance imaging
PMS	Progressive multiple sclerosis
PPMS	Primary progressive multiple sclerosis
SPMS	Secondary progressive multiple sclerosis
RRMS	Relapsing-remitting multiple sclerosis
PIRA	Progression independent of relapse activity
Treg	Regulatory T cell
TCR	T cell receptor
EBV	Epstein–Barr virus
MBP	Myelin basic protein
βsyn	Beta-synuclein
DC	Dendritic cell
S1P	Sphingosine-1-phosphate
BTKi	Bruton’s tyrosine kinase inhibitor
HLA	Human leukocyte antigen
DR15	Human leukocyte antigen allele DRB1:15*01
CNS	Central nervous system
CSF	Cerebrospinal fluid
APC	Antigen presenting cell
EAE	Experimental autoimmune encephalomyelitis
CAR	Chimeric antigen receptor
